# Pigs in sequence space: A 0.66X coverage pig genome survey based on shotgun sequencing

**DOI:** 10.1186/1471-2164-6-70

**Published:** 2005-05-10

**Authors:** Rasmus Wernersson, Mikkel H Schierup, Frank G Jørgensen, Jan Gorodkin, Frank Panitz, Hans-Henrik Stærfeldt, Ole F Christensen, Thomas Mailund, Henrik Hornshøj, Ami Klein, Jun Wang, Bin Liu, Songnian Hu, Wei Dong, Wei Li, Gane KS Wong, Jun Yu, Jian Wang, Christian Bendixen, Merete Fredholm, Søren Brunak, Huanming Yang, Lars Bolund

**Affiliations:** 1 Center for Biological Sequence Analysis, Technical University of Denmark, Lyngby, Denmark; 2 Bioinformatics Research Center, University of Aarhus, Aarhus, Denmark; 3 Division of Genetics, The Royal Veterinary and Agricultural University, Copenhagen, Denmark; 4 Department of Animal Breeding and Genetics, Danish Institute of Agricultural Sciences, Foulum, Denmark; 5 Institute of Human Genetics, University of Aarhus, Aarhus, Denmark; 6 Beijing Genomics Institute, Beijing, China

## Abstract

**Background:**

Comparative whole genome analysis of Mammalia can benefit from the addition of more species. The pig is an obvious choice due to its economic and medical importance as well as its evolutionary position in the artiodactyls.

**Results:**

We have generated ~3.84 million shotgun sequences (0.66X coverage) from the pig genome. The data are hereby released (NCBI Trace repository with center name "SDJVP", and project name "Sino-Danish Pig Genome Project") together with an initial evolutionary analysis.

The non-repetitive fraction of the sequences was aligned to the UCSC human-mouse alignment and the resulting three-species alignments were annotated using the human genome annotation. Ultra-conserved elements and miRNAs were identified. The results show that for each of these types of orthologous data, pig is much closer to human than mouse is. Purifying selection has been more efficient in pig compared to human, but not as efficient as in mouse, and pig seems to have an isochore structure most similar to the structure in human.

**Conclusion:**

The addition of the pig to the set of species sequenced at low coverage adds to the understanding of selective pressures that have acted on the human genome by bisecting the evolutionary branch between human and mouse with the mouse branch being approximately 3 times as long as the human branch. Additionally, the joint alignment of the shot-gun sequences to the human-mouse alignment offers the investigator a rapid way to defining specific regions for analysis and resequencing.

## Background

The domesticated pig (*Sus scrofa*) is an obvious choice for genome sequencing, because of its important economic value for meat production and its relevance to biomedical research. The evolutionary position of the pig as an artiodactyl, where no other large scale sequencing efforts have so far been published, makes it valuable for comparative genomics.

Comparative vertebrate genome analysis – e.g. with the aim of understanding evolutionary pressures on the human sequence – is most cost-effective with relatively low coverage, genome wide sequencing of species at different evolutionary distances [1,2]. The mouse and rat genomes [3,4] and the chimpanzee genome [5] have offered sequences, which are evolutionary quite distant and very close to the human genome sequence, respectively. The evolutionary close chimpanzee sequence can pinpoint significant recent changes in genes, but is not efficient for identification of important regions by comparative approaches because many regions are extremely conserved by chance effects and differences in mutation rates over the genome. The more distantly related rodent sequences are useful in the search for conserved regions of biological importance. However, more species are needed, and an artiodactyl like pig is an obvious choice for the following reasons. 1), By comparing rodents and human, it is not possible to determine whether observable differences, e.g. difference in isochore structure, are mainly due to changes in the rodent or primate lineage since their divergence (approximately 90 million years ago, see 6); 2) Rodents have only a subset of the biological functions important to humans. By including pig additional functions will be covered; 3) Even though rodents, artiodactyls and primates diverged at approximately the same time [6,7], molecular evolution has been faster in the rodent branch, thus the pig is expected to be closer in sequence to human than mouse is. The importance of these points is generally appreciated and comparative genomics sequencing initiatives focusing on restricted regions of the genomes have recently provided much insight [e.g. [1,2]].

The Chinese-Danish pig sequencing consortium has generated about 3.84 million high quality sequences from 5 pig breeds. The present study releases these data and reports an initial evolutionary analysis which confirms that pig and human are closer in sequence space and quantifies the rates of evolution in the pig, rodent and human lineages for various categories of the genome sequences.

## Results

Table 1 shows the amount of high quality sequence obtained from 5 pig breeds (NCBI Trace repository under center name "SDJVP", and project name "Sino-Danish Pig Genome Project"). The average trimmed length of the ~3.84 million sequences was 543 base pairs, yielding a total of 2.1 billion base pairs, equivalent to 0.66X coverage of redundancy of the 3.15 billion base pair pig genome. It is expected that 1-(1-543/3.15 × 10^9^)^3.84 × 10^6 ^= 48% of the pig genome sequence has been hit at least once by this sequencing project. The low coverage prevents making a real assembly of the pig sequences and, thus, the contig coverage is not estimated. The analyses are therefore based on a very large number of short alignments. Repeatmasking (supplementary Table 1) masked 36% of all base pairs. The distribution of repeat types is overall very similar to what is observed in human, except for the expected absence of Alu-elements (Additional file 1). Overall, 38% of the coding fraction of the human-mouse alignment, 38% of the 5' UTR, 33 % of the 3' UTR, 23% of the intron region and 24% of the intergenic region could be expanded to a three-species alignment with the addition of the pig reads. This coverage of the human-mouse alignment by the pig genome sequences was close to our prior expectation. Since only 48% of the base pairs in the pig genome are expected to have been hit, we would only expect to hit at most 48% of the human-mouse alignment, assuming perfect conservation. However, in practice there is some lack of power in BLAST due to the fragmented nature of the pig shotgun reads (being fragmented even more by the repeatmasking), and we expect that some of the human-mouse alignment has no longer an orthologues region in the pig genome. For the non-coding regions, the coverage of the human-mouse alignment by the pig genome sequences is lower than for the coding regions, but this may be explained by lower selective constraints and a much higher rate of insertions-deletions in these regions.

**Table 1:** Overview of the number of raw reads generated from each breed.

**Breed**	**Number of high quality reads**	**Number of bases**
Hampshire	707,281	363,550,668
Yorkshire	1,204,666	652,086,833
Landrace	650,609	342,562,503
Duroc	1,015,722	574,663,060
ErHuaLian	256,993	150,835,661
**Total**	3,835,271	2,083,698,725

**Figure 1:**
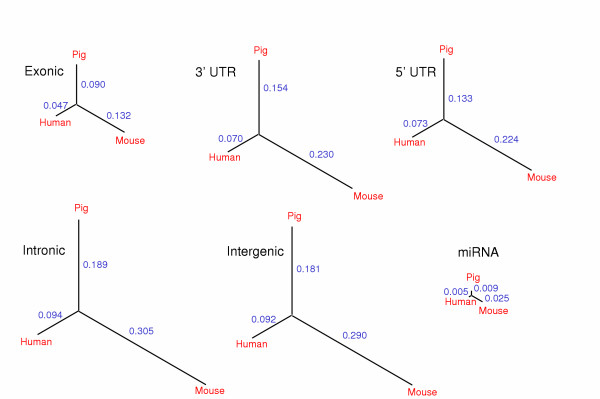
Evolutionary distances between mouse, pig and human for conserved sequences divided into functional classes using the annotation of the human genome. Branch lengths are estimated using the HKY substitution model with gamma correction [12].

The alignments were used to generate the phylogenetic trees in Figure 1. As the pig, mouse and human lineages are believed to have diverged at approximately the same time, the trees allow for separate studies of evolution on the human and mouse branches since the divergence of the two species (the root). Due to a generally lower rate of nucleotide substitutions in the pig and human lineages, the porcine sequences are more similar to the human than to the mouse sequences. Overall, the exonic sequences show the slowest evolution, followed by 5' UTR, 3'UTR, intergenic and intronic regions, reflecting different levels of selective constraint on these domains.

### Ultra-conserved sequences

By aligning the set of ultra-conserved regions against the pig genome reads using BLAST, we were able to find 239 of the 481 known regions reported in Bejerano *et al*. (2004) with a significant hit of at least 150 bp. Only 12 of these regions were less than 98% conserved (85–97% identity). This result agrees very well with the expected 48% of the pig genome being covered and the assumption that these regions are very well conserved within Mammalia.

By aligning the pig shotgun data against all human transcripts (NCBI build 34) we found 758 completely conserved sequences exceeding 200 bp in length. Of these, 41 were also found to be completely conserved in the mouse genome, while 590 were less conserved (more than 95% identity over at least 80% of the length). BLASTing human transcripts vs. the fully assembled mouse genome (NCBI build 32), we found 2709 ultra-conserved regions. When aligning this set of sequences against the artificially fragmented mouse genomic dataset using BLAST it was only possible to classify 664 (24.5%) as ultra-conserved – less than the 758 elements found in the human-pig comparison.

### miRNA

The set of pig miRNAs (Additional file 2) was compared to human and mouse and it was possible to obtain 50 three-way alignments. The evolutionary tree in Figure 1 was constructed using the HKY+gamma model from these alignments with gap positions removed. By construction, the miRNAs are more conserved than even the protein coding sequences, but with pig and human being phylogenetically closest. For the 50 triple-alignments, we obtained 25 cases where pig is closer to human than to mouse, 2 cases where pig is closer to mouse than to human, and 23 cases where pig is equally distant to human and mouse.

### GC content

**Figure 2:**
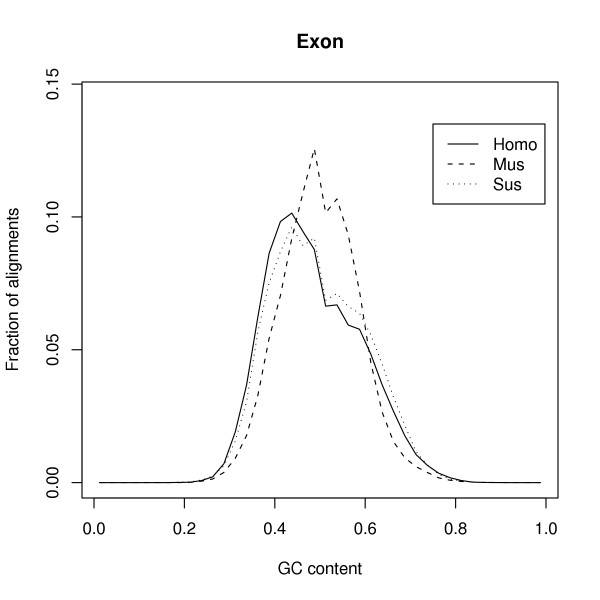
The distribution of GC content in exons for human, pig and mouse. Only alignments with more than 40 base pairs of exon sequence were used.

The intra-genomic variation in GC content among the individual alignments reflects the isochore structure of the genome. Thus, from the three species alignments, we calculated the GC content for each functional sequence class for each aligned fragment. For a given type of sequence, only alignments having more than 40 nucleotides of the specific type were used. Table 2 shows that the mean GC content is similar among the three species. The variance among alignments in GC content is generally lower in mouse than in pig and human, but mostly so for coding sequences, followed by the UTR and intron regions (Table 2). Figure 2 shows the distribution of GC% for the coding alignments. The reduced variability in GC content in mouse compared to human has been shown previously, e.g. Figure 8a in [4]. The results presented here suggest a very similar pattern in human and pig.

**Table 2:** Average GC content and the variance among alignments exceeding 40 bp for each species and each functional category. Variance is standardized to the variance observed in the human sequence.

	**Mean GC content**			**Variance GC content**		
**Type of sequence**	**Human**	**Mouse**	**Pig**	**Human**	**Mouse**	**Pig**
Intron	0.390	0.413	0.407	1	0.82	1.02
Coding	0.487	0.500	0.496	1	0.69	1.01
3' UTR	0.404	0.426	0.418	1	0.77	1.03
5' UTR	0.595	0.593	0.592	1	0.81	0.92
Intergenic	0.384	0.399	0.396	1	0.91	1.01

## Discussion

Even though divergence between pig and human occurred approximately at the same time as the divergence between human and mouse, the pig sequence is much more similar to the human sequence. Thus, the availability of the pig sequence effectively subdivides the human-mouse evolutionary branch at a position closest to human. This implies that one can determine which changes occurred on the human and mouse branches, respectively, since their divergence. The results of the phylogenetic analysis show that the relative length of the mouse, human and pig branches are different for the different types of data. Intronic sites and intergenic sites show a similar pattern, which also closely resembles that of synonymous sites [see [7]], reflecting that purifying selection is probably weak and similar for these regions. It is noteworthy that intergenic branch lengths are slightly shorter than intronic ones (and both are shorter than synonymous sites, see [7]). This may reflect either 1) more selective constraints on intergenic sequences than intronic, i.e. parts of the annotated intergenic sequences are indeed genic, or 2) a bias in the construction of the human-mouse alignment that make it easier to align sequence close to the conserved exons so that somewhat more divergent intronic sequence can be detected. The differences in evolutionary rates among the three species are most likely attributable to differences in generation times since they diverged. Non-synonymous sites show shorter branches that are much more similar in lengths among the species [7]. This reflects purifying selection, which has been strongest in the mouse lineage, followed by pig and then human [20,21]. The reason for this may be the larger average population size in the mouse since divergence. The UTR regions shows more selective constraint than introns.

The similarity between human and pig adds to the recently reported ultra-conserved regions [14]. Pig and human share more ultra-conserved regions than human and mouse, and (correcting for the coverage in the data presented) virtually all of the ultra-conserved elements defined by Bejerano *et al*. [14] are also found in pig and therefore most likely in the artiodactyls lineage.

Variation in GC content along the genome (isochore structure) is more pronounced in primates than in rodents [4,5]. The present results extend these findings and put artiodactyls in line with primates – lending further support to the suggestion that isochore evolution in rodents deviates from all other lineages, possibly because of extensive genome rearrangements [22].

## Conclusion

A 0.66X coverage pig genome survey is hereby released. Even though it is only a beginning, the data offer many analytical possibilities and should also stimulate the international initiatives to generate a complete draft of the pig genome. The initial analysis of the data adds to our understanding of the evolutionary relationships of humans, mice and pigs. Further comparative genomic studies and more detailed genetic analyses will greatly improve our ability to elucidate pig as well as human biology and medicine.

## Methods

### Generation of pig shot-gun sequences

Genomic DNA was extracted from blood samples from 5 different breeds of *Sus scrofa *(domestic pig): ErHuaLian, Duroc, Landrace, Yorkshire and Hampshire. Following mechanical shearing, DNA fragments (1–3 kb) were isolated by gel electrophoresis and cloned into the SmaI restriction site of the pUC18 plasmid using blunt-end ligation. After transformation into *E. coli *(strain DH5a) and selection on LB-plates, individual clones were picked for the library. The quality of a library was checked by sequencing a small number of plasmids, which were assembled by phrap and aligned by BLAST to validate the randomness of the library and the proportion of contaminations – including mitochondrial DNA, human fragments and vector sequences. If the library was eligible, plasmid DNA was extracted from the individual clones using the membrane-filter method (Millipore). The pig genomic inserts were sequenced using the M13 bidirectional sequencing primers on the MegaBACE1000 platform using ET dye terminator. The chromatograms were registered in a relational database tracking all generated data to eliminate duplicated work and check for errors. Using phred v. 0.020425.c with a quality cut-off of 0.05 and the -trim_alt parameter the chromatograms were traced and the resulting traces were masked for vector sequences using CROSSMATCH (0.990329). Resulting sequences were resubmitted to the relational database and prepared for submission. Following removal of contamination from vector and bacterial host, we retained 3,835,271 reads of at least 150 bp.

All sequences with trace files have been submitted to the Ensembl/NCBI Trace repository under the center name "SDJVP", and project name "Sino-Danish Pig Genome Project".

### Repetitive elements

Standard masking of repetitive sequences was performed using Repeatmasker version 2004/03/06 with RepBase Update 8.12 with *Sus scrofa *as query species, using default settings.

### Construction of three species alignments

The newest build of the human-mouse pairwise alignment (hg17/mm5) based on the improved blastZ algoritm [8] was downloaded from the UCSC genome browser [9] and the repeatmasked shotgun-sequences were BLAST'ed up against this alignment using Megablast [10] with the following settings: (w) word size = 12, (e) minimum e-value = 0.0001, (x) extension parameter = 50, (u) Repeatmasked query sequences = True. Resulting hits were then used as tags to build the alignment around (see below).

All query sequences with multiple blast hits on different parts of the human-mouse alignment (approximately 10%) were removed at this point and only the remaining pig sequences were used in the further analyses. This was done to conservatively eliminate paralogous hits. The individual blast hits were used to position the pig query sequences on a specific part of the human-mouse alignment. For each BLAST hit a region extending 300 base pairs in both directions was then realigned using DIALIGN version 2.2.1 with default parameters [11]. Regions that afterwards were not considered by DIALIGN to be aligned were removed at this point. Subsequently an annotation file (refGene.txt) of the human genome (hg17) was downloaded from the UCSC genome server and used to annotate each position in the alignment according to the following classes 1) protein-coding exon (with reading frame position), 2) intron, 3) 5' UTR, 4) 3' UTR, and 5) intergenic. Phylogenetic analyses were performed on each of these classes separately.

### Estimation of phylogenetic trees

A combined alignment for each of the five functional classes was constructed by concatenating the many small three species alignments, and gap positions were removed. For each class of data (exon, 5'UTR, 3'UTR, intron and intergenic), the implementation of the HKY85+GAMMA model [12] in PAML v. 3.14 [13] was used to estimate the number of substitutions in each evolutionary branch.

### Analysis of ultra-conserved regions

The data set containing the 481 ultra-conserved regions defined by Bejerano *et al*. [14] was downloaded and used for the first part of this analysis. We estimated how many of these regions that can be found in the pig genome shotgun sequences by a simple BLAST (blastn) approach retaining only hits of a length of 150 bp or more. Since these ultra-conserved regions are based on external data, artefacts due to the relatively low coverage of the pig genome sequences are not important here.

In the second part of the analysis (searching for novel ultra-conserved regions), it was important to address the artefacts that arise from searching for ultra conserved regions in a fragmented dataset. In order to do a comparable search for ultra-conserved regions against the mouse and pig genomes, we artificially created a mouse data set resembling the fragmented pig shotgun data set. Since the identification criteria we use is 100% conservation over 200 bp or more, the length of the individual read as well as the quality become a major factor. The fragmented mouse data set was generated by downloading all ~79 million reads of the NCBI trace database, shuffle these randomly, and pick single reads until the same amount of nucleotides was reached as in the pig genomic reads dataset. Only pig reads with length > 200 bp were used and the data set consisted of 2,034,999,640 bp from pig and 2,665,153 reads (2,034,999,649 bp) from mouse.

### Construction of the miRNA data set

The reads were BLAST searched [15] (default options) against the miRNA hairpin database [16] and matches of at least 60 nucleotides clustered. The obtained set of 219 clusters contained redundancy as the same pig reads hit the same miRNAs from different species (human, mouse, etc.). This could be converted into a unique set of 68 clusters (each containing 2–5 reads), i.e. each type of miRNA is now only represented once. Each cluster was assembled with Cap3 [17] from TGICL [18]. As a result 84 contigs and singletons were obtained and BLAST searched back against the miRNA database. Matches with more than 95% identity in an alignment length larger than 95% of the miRNA hairpin length were selected. This resulted in 54 hairpins complying with the criteria defined in Ambros et al. [19]. In total 51 different mature sequences are covered in the 54 hairpins.

## Authors' contributions

LB, HY, CB, MF, SB initiated and coordinated the project, JUW, BL, SH, WD, WL, JY, JIW, HY organized the sequencing work, FP, HHS, HH, AK cleaned up the data and created the database, RW, MHS, FGJ, JG, OFC, TM performed the analyses, RW, MHS, FG, OFC, JUW, GW, LB drafted the paper. All authors have read and approved the paper.

## Supplementary Material

Additional File 1 Distribution of repetitive elements in the pig genome survey sequences. Data obtained from Repeatmasking of shotgun sequences Click here for file

Additional File 2 Details of investigated miRNAs. Click here for file
