# Supplementary material for: Pigs in sequence space: A 0.66X coverage pig genome survey based on shotgun sequencing
Source: BMC Genomics. 2005 May 10;6:70. doi: 10.1186/1471-2164-6-70 (PMC1142312; doi:10.1186/1471-2164-6-70)
Supplement: Additional File 1 — Distribution of repetitive elements in the pig genome survey sequences. Data obtained from Repeatmasking of shotgun sequences [file 1471-2164-6-70-S1.doc]

**Additional file 1:**

**Additional Table 1**: Distribution of repetitive elements in the pig genome survey sequences.

|  | Number of elements | Total number of bases (bp) | Fraction of sequence |
| --- | --- | --- | --- |
| SINEs: | 1,445,114 | 235,501,025 | 11.30% |
| MIRs: | 288,209 | 36,548,068 | 1.75% |
| LINEs: | 1,169,150 | 336,411,059 | 16.14% |
| LINE1: | 970,306 | 301,948,407 | 14.49% |
| LINE2: | 177,673 | 30,967,877 | 1.49% |
| L3/CR1: | 21,171 | 3,494,775 | 0.17% |
| LTR elements: | 264,871 | 58,415,010 | 2.80% |
| MaLRs: | 144,953 | 31,268,067 | 1.50% |
| ERVL: | 68,638 | 16,048,615 | 0.77% |
| ERV_classI: | 43,659 | 9,804,121 | 0.47% |
| DNA elements: | 193,399 | 31,514,664 | 1.51% |
| MER1_type: | 120,602 | 18,707,397 | 0.90% |
| MER2_type: | 30,907 | 5,952,609 | 0.29% |
| Unclassified: | 627 | 102,771 | 0.00% |
| Small RNA: | 7,135 | 485,842 | 0.02% |
| Satellites: | 94,820 | 30,607,333 | 1.47% |
| Simple repeats: | 303,026 | 12,902,607 | 0.62% |
| Low complexity: | 294,623 | 11,033,980 | 0.53% |
