# Supplementary material for: Pigs in sequence space: A 0.66X coverage pig genome survey based on shotgun sequencing
Source: BMC Genomics. 2005 May 10;6:70. doi: 10.1186/1471-2164-6-70 (PMC1142312; doi:10.1186/1471-2164-6-70)
Supplement: Additional File 2 — Details of investigated miRNAs. [file 1471-2164-6-70-S2.doc]

**Additional file 2**

**Additional Table 2.** Details of investigated miRNAs.

| **Mature name** | **Mature sequence** | **Hairpin name** | **Hairpin sequence** | **Mature name** | **Mature sequence** | **Hairpin name** |
| --- | --- | --- | --- | --- | --- | --- |
| miR-ssc-105 | UCAAAUGCUCAGACUCCUGU | mir-ssc-105-1 | ugugcaucguagUCAAAUGCUCAGACUCCUGUgguggcugcucaugcaccacggauguuugagcaugugcuacggugucua | hsa-miR-105 | UCAAAUGCUCAGACUCCUGU | hsa-mir-105-1 |
| miR-ssc-105* | UCAAAUGCUCAGACUCCUUG | mir-ssc-105-2* | ugugcaucguggUCAAAUGCUCAGACUCCUUGguggcugcuuaugcaccacggauguuugagcaugugcuauggugucua | hsa-miR-105 | UCAAAUGCUCAGACUCCUGU | hsa-mir-105-2 |
| miR-ssc-106a | AAAAGUGCUUACAGUGCAGGUAGC | mir-ssc-106a | ccuuggccguguAAAAGUGCUUACAGUGCAGGUAGCuuuuugagaucuacugcaaugcaagcacuucuuacauuaccaugg | hsa-miR-106a | AAAAGUGCUUACAGUGCAGGUAGC | hsa-mir-106a |
| miR-ssc-122a | UGGAGUGUGACAAUGGUGUUUGU | mir-ssc-122a | ccuuagcagagcugUGGAGUGUGACAAUGGUGUUUGUguccaaacuaucaaacgccauuaucacacuaaauagcuacuguuaggc | hsa-miR-122a | UGGAGUGUGACAAUGGUGUUUGU | hsa-mir-122a |
| miR-ssc-125b | UCCCUGAGACCCUAACUUGUGA | mir-ssc-125b-2 | accagacuuuuccuagUCCCUGAGACCCUAACUUGUGAgguauuuuaguaacaucacaagucaggcucuugggaccuaggcggagggga | hsa-miR-125b | UCCCUGAGACCCUAACUUGUGA | hsa-mir-125b-2 |
| miR-ssc-135a | UAUGGCUUUUUAUUCCUAUGUGA | mir-ssc-135a-1 | aggccucgcuguucucUAUGGCUUUUUAUUCCUAUGUGAuucuacugcucauucauauagggauuggagccguggcgcacggcggggaca | hsa-miR-135a | UAUGGCUUUUUAUUCCUAUGUGA | hsa-mir-135a-1 |
| miR-ssc-135a | UAUGGCUUUUUAUUCCUAUGUGA | mir-ssc-135a-2 | agauaaauucacucuagugcuuUAUGGCUUUUUAUUCCUAUGUGAuaguaauaaagucucauguagggauggaagccaugaaauacauugugaaaaauca | hsa-miR-135a | UAUGGCUUUUUAUUCCUAUGUGA | hsa-mir-135a-2 |
| miR-ssc-145 | GUCCAGUUUUCCCAGGAAUCCCUU | mir-ssc-145 | caccuuguccucacgGUCCAGUUUUCCCAGGAAUCCCUUagaugcugagauggggauuccuguaaauacuguucuugaggucaugg | hsa-miR-145 | GUCCAGUUUUCCCAGGAAUCCCUU | hsa-mir-145 |
| miR-ssc-148a | UCAGUGCACUACAGAACUUUGU | mir-ssc-148a | gaggcaaaguucugagacacuccgacucugaauaugauagaagUCAGUGCACUACAGAACUUUGUcuc | hsa-miR-148a | UCAGUGCACUACAGAACUUUGU | hsa-mir-148a |
| miR-ssc-15b* | CCGCAGCACAUCAUGGUUUACA | mir-ssc-15b* | uugaggccuuaaaguacugCCGCAGCACAUCAUGGUUUACAuacuacaaucaagaugcgaaucauuauuugcugcucuagaaauuuaaggaaauucau | hsa-miR-15b | UAGCAGCACAUCAUGGUUUACA | hsa-mir-15b |
| miR-ssc-181b | AACAUUCAUUGCUGUCGGUGGGUU | mir-ssc-181b-2 | auggcugcacucAACAUUCAUUGCUGUCGGUGGGUUugagucugaaucaacucacugaucaaugaaugcaaacugcggaccaaaca | hsa-miR-181b | AACAUUCAUUGCUGUCGGUGGGUU | hsa-mir-181b-2 |
| miR-ssc-184 | UGGACGGAGAACUGAUAAGGGU | mir-ssc-184 | ccagucacauccccuuaucacuuuuccagccagcuuugugacucuaaauguUGGACGGAGAACUGAUAAGGGUaggugauuga | hsa-miR-184 | UGGACGGAGAACUGAUAAGGGU | hsa-mir-184 |
| miR-ssc-19a | UGUGCAAAUCUAUGCAAAACUGA | mir-ssc-19a | gcaguccucuguuaguuuugcauaguugcacuacaagaagaauguaguUGUGCAAAUCUAUGCAAAACUGAugguggccugc | hsa-miR-19a | UGUGCAAAUCUAUGCAAAACUGA | hsa-mir-19a |
| miR-ssc-20 | UAAAGUGCUUAUAGUGCAGGUA | mir-ssc-20 | guagcacUAAAGUGCUUAUAGUGCAGGUAguguuuaguuaucuacugcauuaugagcacuuaaaguacugc | hsa-miR-20 | UAAAGUGCUUAUAGUGCAGGUA | hsa-mir-20 |
| miR-ssc-216 | UAAUCUCAGCUGGCAACUGUG | mir-ssc-216 | gauggcugugaguuggcuUAAUCUCAGCUGGCAACUGUGagauguucauacaaucccccacaguggucucugggauuaugcuaaacagagcaauuuccuugcccu | hsa-miR-216 | UAAUCUCAGCUGGCAACUGUG | hsa-mir-216 |
| miR-ssc-217 | UACUGCAUCAGGAACUGAUUGGAU | mir-ssc-217 | auaauuauuacagaguuuuugaugucgcagaUACUGCAUCAGGAACUGAUUGGAUaagaauuggucaccaucaguucuuaaugcauugccuucagcaucuaaacaag | hsa-miR-217 | UACUGCAUCAGGAACUGAUUGGAU | hsa-mir-217 |
| miR-ssc-224 | CAAGUCACUAGUGGUUCCGUUUA | mir-ssc-224 | gggcuuuCAAGUCACUAGUGGUUCCGUUUAguagaagguuuugcauuguuucaaaauggugcccuagugacuacaaagccc | hsa-miR-224 | CAAGUCACUAGUGGUUCCGUUUA | hsa-mir-224 |
| miR-ssc-23a | AUCACAUUGCCAGGGAUUUCC | mir-ssc-23a | cggcugggguuccuggggaugggauuugcugccugucacaaAUCACAUUGCCAGGGAUUUCCaaucgacc | hsa-miR-23a | AUCACAUUGCCAGGGAUUUCC | hsa-mir-23a |
| miR-ssc-24 | UGGCUCAGUUCAGCAGGAACAG | mir-ssc-24-2 | cucugccucccgugccuacugagcugaaacacaguugauuugugcagacUGGCUCAGUUCAGCAGGAACAGg | hsa-miR-24 | UGGCUCAGUUCAGCAGGAACAG | hsa-mir-24-2 |
| miR-ssc-26a | UUCAAGUAAUCCAGGAUAGGCU | mir-ssc-26a-2 | ggcuguggcuggaUUCAAGUAAUCCAGGAUAGGCUguuuccaucugugaggccuauucuugauuacuuguuucuggaggcagcu | hsa-miR-26a | UUCAAGUAAUCCAGGAUAGGCU | hsa-mir-26a-2 |
| miR-ssc-28* | AAGGAGCUCACACUCUAUUGAG | mir-ssc-28* | gguccuugcccucAAGGAGCUCACACUCUAUUGAGuugccuuucugucuuccccacuagauugugagcuccuggagggcaggcacu | hsa-miR-28 | AAGGAGCUCACAGUCUAUUGAG | hsa-mir-28 |
| miR-ssc-29b | UAGCACCAUUUGAAAUCAGU | mir-ssc-29b-1 | cuucaggaagcugguuucauauggugguuuagauuuaaaaagugauugucUAGCACCAUUUGAAAUCAGUguucuuggggg | hsa-miR-29b | UAGCACCAUUUGAAAUCAGU | hsa-mir-29b-1 |
| miR-ssc-301* | CAGUCCAAUAGUAUUGUCAAAGC | mir-ssc-301* | gcuaacaaaugcucugacuuuauugcacuacuguacuuuacagcuacCAGUCCAAUAGUAUUGUCAAAGCaucugagagcag | hsa-miR-301 | CAGUGCAAUAGUAUUGUCAAAGC | hsa-mir-301 |
| miR-ssc-323 | GCACAUUACACGGUCGACCUCU | mir-ssc-323 | uugguacuuggagagaggugguccguggcgcguucgcuuuauuuauggcGCACAUUACACGGUCGACCUCUuugcgguaucuaauc | hsa-miR-323 | GCACAUUACACGGUCGACCUCU | hsa-mir-323 |
| miR-ssc-326 | CCUCUGGGCCCUUCCUCCAG | mir-ssc-326 | ucugucuguugggcuggaggcagggccuuugugcaggcggguugugcucagaucgCCUCUGGGCCCUUCCUCCAGcccagaggcggauuca | hsa-miR-326 | CCUCUGGGCCCUUCCUCCAG | hsa-mir-326 |
| miR-ssc-7 | UGGAAGACUAGUGAUUUUGUU | mir-ssc-7-1 | uggauguuggucuaguucugugUGGAAGACUAGUGAUUUUGUUguuuuuagauaacuaaaucgacaacaaaucacagucugccauauggcacaggccaugccucuacag | hsa-miR-7 | UGGAAGACUAGUGAUUUUGUU | hsa-mir-7-1 |
| miR-ssc-95 | UUCAACGGGUAUUUAUUGAGCA | mir-ssc-95 | aacacagugggcgcucaauaaauguuuguugaauugagaugcguuaaaUUCAACGGGUAUUUAUUGAGCAcccacucugug | hsa-miR-95 | UUCAACGGGUAUUUAUUGAGCA | hsa-mir-95 |
| miR-ssc-140 | AGUGGUUUUACCCUAUGGUAG | mir-ssc-140 | ccugccAGUGGUUUUACCCUAUGGUAGguuacgucaugcuguucuaccacaggguagaaccacggacagg | mmu-miR-140 | AGUGGUUUUACCCUAUGGUAG | mmu-mir-140 |
| miR-ssc-181c | AACAUUCAACCUGUCGGUGAGU | mir-ssc-181c | caaggguuugggggAACAUUCAACCUGUCGGUGAGUuugggcagcucaggcaaaccaucgaccguugaguggaccccgaggccugga | mmu-miR-181c | AACAUUCAACCUGUCGGUGAGU | mmu-mir-181c |
| miR-ssc-183 | UAUGGCACUGGUAGAAUUCACUG | mir-ssc-183 | cugugUAUGGCACUGGUAGAAUUCACUGugaacagucucggucagugaauuaccgaagggccauaaacag | mmu-miR-183 | UAUGGCACUGGUAGAAUUCACUG | mmu-mir-183 |
| miR-ssc-205 | UCCUUCAUUCCACCGGAGUCUG | mir-ssc-205 | cucuugUCCUUCAUUCCACCGGAGUCUGucucauacccaaccagauuucaguggagugaagcucaggag | mmu-miR-205 | UCCUUCAUUCCACCGGAGUCUG | mmu-mir-205 |
| miR-ssc-214 | ACAGCAGGCACAGACAGGCAG | mir-ssc-214 | ggccuggcuggacggaguugucaugugucugccugucuacacuugcugugcagaacauccgcucaccuguACAGCAGGCACAGACAGGCAGucacaugacaacccagccu | mmu-miR-214 | ACAGCAGGCACAGACAGGCAG | mmu-mir-214 |
| miR-ssc-27a | UUCACAGUGGCUAAGUUCCGC | mir-ssc-27a | uggccuggggagcagggcuuagcugcuugugagcagguccacagcaagucgugUUCACAGUGGCUAAGUUCCGCccccugga | mmu-miR-27a | UUCACAGUGGCUAAGUUCCGC | mmu-mir-27a |
| miR-ssc-32 | UAUUGCACAUUACUAAGUUGC | mir-ssc-32 | ggagaUAUUGCACAUUACUAAGUUGCauguugucacggccucagugcaauuuagugugugugauauuuuc | mmu-miR-32 | UAUUGCACAUUACUAAGUUGC | mmu-mir-32 |
| miR-ssc-325* | CCUAGUAGGUGUUCAGUAAGUGU | mir-ssc-325* | agugcuugguuCCUAGUAGGUGUUCAGUAAGUGUuugugacauaauucguuuauugagcaccuccuaucaaucaagcacugugcuagguucugg | mmu-miR-325 | CCUAGUAGGUGCUCAGUAAGUGU | mmu-mir-325 |
| miR-ssc-7c | UGAGGUAGUAGGUUGUAUGGUU | mir-ssc-7c-1 | ugugugcauccggguUGAGGUAGUAGGUUGUAUGGUUuagaguuacaccgugggaguuaacuguacaaccuucuagcuuuccuuggagcacacu | rno-let-7c | UGAGGUAGUAGGUUGUAUGGUU | rno-let-7c-1 |
| miR-ssc-7f | UGAGGUAGUAGAUUGUAUAGUU | mir-ssc-7f-2 | ugugggaUGAGGUAGUAGAUUGUAUAGUUuuagggucauaccccaucuuggagauaacuauacagucuacugucuuucccacg | rno-let-7f | UGAGGUAGUAGAUUGUAUAGUU | rno-let-7f-2 |
| miR-ssc-7i | UGAGGUAGUAGUUUGUGCU | mir-ssc-7i | cuggcUGAGGUAGUAGUUUGUGCUguuggucggguugugacauugcccgcuguggagauaacugcgcaagcuacugccuugcuag | rno-let-7i | UGAGGUAGUAGUUUGUGCU | rno-let-7i |
| miR-ssc-103 | AGCAGCAUUGUACAGGGCUAUGA | mir-ssc-103-1 | cuuacugcccucggcuucuuuacagugcugccuuguugcauauggaucaAGCAGCAUUGUACAGGGCUAUGAaggcacugag | rno-miR-103 | AGCAGCAUUGUACAGGGCUAUGA | rno-mir-103-1 |
| miR-ssc-107 | AGCAGCAUUGUACAGGGCUAUCA | mir-ssc-107 | uucucucugcuuucagcuucuuuacaguguugccuuguggcauggaguucaAGCAGCAUUGUACAGGGCUAUCAaagcacagagagc | rno-miR-107 | AGCAGCAUUGUACAGGGCUAUCA | rno-mir-107 |
| miR-ssc-124a | UUAAGGCACGCGGUGAAUGCCA | mir-ssc-124a-1 | aggccucucucuccguguucacagcggaccuugaguuaaauguccauacaaUUAAGGCACGCGGUGAAUGCCAagaauggggcug | rno-miR-124a | UUAAGGCACGCGGUGAAUGCCA | rno-mir-124a-1 |
| miR-ssc-128a | UCACAGUGAACCGGUCUCUUUU | mir-ssc-128a | ugagcuguuggauucggggccguagcacugucugagagguuuacauuucUCACAGUGAACCGGUCUCUUUUucagcugcuuc | rno-miR-128a | UCACAGUGAACCGGUCUCUUUU | rno-mir-128a |
| miR-ssc-136 | ACUCCAUUUGUUUUGAUGAUGGA | mir-ssc-136 | ugagcccucggaggACUCCAUUUGUUUUGAUGAUGGAuucuuacgcuccaucaucgucucaaaugagucuucagaggguucu | rno-miR-136 | ACUCCAUUUGUUUUGAUGAUGGA | rno-mir-136 |
| miR-ssc-139 | UCUACAGUGCACGUGUCU | mir-ssc-139 | guguauUCUACAGUGCACGUGUCUccaguguggcucggaggcuggagacgcaggccauguuggaguaac | rno-miR-139 | UCUACAGUGCACGUGUCU | rno-mir-139 |
| miR-ssc-153 | UUGCAUAGUCACAAAAGUGA | mir-ssc-153 | agcgguggccagugucauuuuugugauguugcagcuaguaauaugagcccagUUGCAUAGUCACAAAAGUGAucauuggaaacugug | rno-miR-153 | UUGCAUAGUCACAAAAGUGA | rno-mir-153 |
| miR-ssc-18 | UAAGGUGCAUCUAGUGCAGAUA | mir-ssc-18 | gugcuuuuuguucUAAGGUGCAUCUAGUGCAGAUAgugaaguagauuagcaucuacugcccuaagugcuccuucuggcauaagaaguuaugu | rno-miR-18 | UAAGGUGCAUCUAGUGCAGAUA | rno-mir-18 |
| miR-ssc-186 | CAAAGAAUUCUCCUUUUGGGCUU | mir-ssc-186 | ugcuuauaacuuucCAAAGAAUUCUCCUUUUGGGCUUuaugauuuuauuuuaagcccaaaggugaauuuuuugggaaguuug | rno-miR-186 | CAAAGAAUUCUCCUUUUGGGCUU | rno-mir-186 |
| miR-ssc-196 | UAGGUAGUUUCAUGUUGUUGG | mir-ssc-196 | uugcucagcugaucuguggcuUAGGUAGUUUCAUGUUGUUGGgauugaguuuugaacucggcaacaagaaacugccugaguuacaucagucgguuaucgucgagggc | rno-miR-196 | UAGGUAGUUUCAUGUUGUUGG | rno-mir-196 |
| miR-ssc-204 | UUCCCUUUGUCAUCCUAUGCCU | mir-ssc-204 | ggcuacaguccuucucaugugacucguggacUUCCCUUUGUCAUCCUAUGCCUgagaauauaugaaggaggcugggaaggcaaagggacguucaauugucaucacuggc | rno-miR-204 | UUCCCUUUGUCAUCCUAUGCCU | rno-mir-204 |
| miR-ssc-21 | UAGCUUAUCAGACUGAUGUUGA | mir-ssc-21 | uguaccaccuugucgggUAGCUUAUCAGACUGAUGUUGAcuguugaaucucauggcaacagcagucgaugggcugucugacauuuugguauc | rno-miR-21 | UAGCUUAUCAGACUGAUGUUGA | rno-mir-21 |
| miR-ssc-29c | UAGCACCAUUUGAAAUCGGUUA | mir-ssc-29c | aucucuuacacaggcugaccgauuucuccugguguucagagucuguuuuugucUAGCACCAUUUGAAAUCGGUUAugauguangggga | rno-miR-29c | UAGCACCAUUUGAAAUCGGUUA | rno-mir-29c |
| miR-ssc-30c | UGUAAACAUCCUACACUCUCAGC | mir-ssc-30c-2 | gacagauacUGUAAACAUCCUACACUCUCAGCuguggaaaguaagaaagcugggagaaggcuguuuacucucucugccuu | rno-miR-30c | UGUAAACAUCCUACACUCUCAGC | rno-mir-30c-2 |
| miR-ssc-9 | UCUUUGGUUAUCUAGCUGUAUGA | mir-ssc-9-1 | cgggguugguuguuaUCUUUGGUUAUCUAGCUGUAUGAgugguguggagucuucauaaagcuagauaaccgaaaguaaaaauaacccca | rno-miR-9 | UCUUUGGUUAUCUAGCUGUAUGA | rno-mir-9-1 |
| miR-ssc-9 | UCUUUGGUUAUCUAGCUGUAUGA | mir-ssc-9-2 | ggaagcgaguuguuaUCUUUGGUUAUCUAGCUGUAUGAguguauuggucuucauaaagcuagauaaccgaaaguaaaaacuccuuca | rno-miR-9 | UCUUUGGUUAUCUAGCUGUAUGA | rno-mir-9-2 |

**Table footnotes**

The 51 miRNA sequence (54 hairpins) in the porcine genome found by sequence similarity to closely related species. Names containing a "*" indicate a slight mismatch between the proposed mature pig sequence and homologue mature sequence. Column 1 gives the name for pig miRNA sequence; column 2 the mature sequence; column 3 the hairpin name; column 4 the hairpin sequence where upper case letters indicate the mature sequence; column 5 the name of the mature homologue; column 6 the sequence of the mature homologue; column 7 the name of the mature hairpins sequence.

Note: In construction of the data set it was tested whether the longer human hairpins gave biased conclusion compared to the shorter mouse sequences. When considering equal length human and mouse hairpins a similar tree was obtained. Also, as miR-105 and miR-95 only exist in human and miR-325 only in mouse (and rat) in the miRNA registry, these miRNAs were discarded from the analysis. By inspection of the BLAST result involving the region corresponding mir-ssc-196, this miRNA was annotated from the rat sequence. However, matches to human (hsa-mir-196-1) and mouse (mmu-mir-196-1) are very close, whereas the matches to the "-2" versions of mir-196 have limited lengths. Therefore a triplet of mir-ssc-196, hsa-mir-196-1 and mmu-mir-196-1 was constructed. Also a BLAST inspection of the pig read containing mir-ssc-153 revealed that it had been annotated from rat (closest match), but that hsa-mir-153-2 and mmu-mir-153 also met the cut-offs mentioned above. Even though hsa-mir-153-1 match is almost full length the sequence similarity is only 90%. Hence a triplet of mir-ssc-153, hsa-mir-153-2 and mmu-mir-153 was used. A similar type of observation was made for mir-ssc-7c-1 (which was annotated from rat) resulting in a triplet of mir-ssc-7c-1, mmu-let-7c-1 and hsa-let-7c.
